# Spatial memory retrieval under cueing in behavioral frontotemporal dementia and Alzheimer’s disease

**DOI:** 10.1590/1980-5764-DN-2025-0413

**Published:** 2026-03-23

**Authors:** Antônio Jaeger, Eduarda Xavier Carreira, Gabriel Gomide, Natália Araújo Sundfeld da Gama, Morgana Cavalcante de Queiroz, Paulo Caramelli, Lucas Porcello Schilling, Leonardo Cruz de Souza

**Affiliations:** 1 Universidade Federal de Minas Gerais, Belo Horizonte MG, Brazil.; 2 Pontifícia Universidade Católica de Minas Gerais, Belo Horizonte MG, Brazil.; 3 Pontifícia Universidade Católica do Rio Grande do Sul, Porto Alegre RS, Brazil.

**Keywords:** Spatial Memory, Memory, Episodic, Dementia, Frontotemporal Dementia, Alzheimer’s Disease, Executive Function, Memória Espacial, Memória Episódica, Demência, Demência Frontotemporal, Doença de Alzheimer, Função Executiva

## Abstract

Recent studies indicate that episodic memory is impaired in behavioral variant frontotemporal dementia (bvFTD), with deficits as severe as those seen in Alzheimer’s disease (AD). Also, both bvFTD and AD patients exhibit executive dysfunction, but the extent to which such dysfunction contributes to memory impairments remains unclear.

**Objective:**

The aim of this study was to investigate interactions between executive functions and episodic memory in AD and bvFTD by manipulating executive control in a spatial source memory task.

**Methods:**

Twenty bvFTD patients, 18 AD patients, and 20 healthy controls completed a source memory test in which they should remember whether objects were previously seen on the left or right side of a computer screen. Critically, arrow cues (66.7% valid, 33.3% invalid) indicated the likely prior location of each object during retrieval.

**Results:**

bvFTD and AD patients showed overall similar memory performances but were impaired compared to controls. While cueing affected memory performances of controls and bvFTD patients, AD patients showed undistinguishable performances after valid and invalid cueing.

**Conclusion:**

These findings support the notion that episodic memory deficits in bvFTD can be as severe as in AD and demonstrate that, unlike bvFTD patients, patients with AD show difficulties in using information conveyed by cues during memory judgments, probably because of underlying attentional deficits.

## RESUMO.

Estudos recentes indicam que a memória episódica está comprometida na demência frontotemporal variante comportamental (bvFTD), com déficits tão graves quanto os observados na doença de Alzheimer (DA). Pacientes com bvFTD e DA também exibem disfunção executiva, mas não está claro em que medida esses prejuízos contribuem para as dificuldades de memória.

**Objetivo:**

Investigar as interações entre funções executivas e memória episódica em pacientes com DA e bvFTD, por meio da manipulação do controle executivo em uma tarefa de memória de fonte espacial.

**Métodos:**

Vinte pacientes com bvFTD, 18 com DA e 20 controles saudáveis realizaram um teste de memória de fonte espacial, em que deviam indicar se os objetos haviam sido apresentados no lado esquerdo ou direito da tela do computador. Durante a fase de recuperação, pistas na forma de setas (66,7 % válidas; 33,3 % inválidas) sinalizavam a provável localização prévia de cada objeto.

**Resultados:**

Pacientes com bvFTD e DA tiveram desempenhos semelhantes entre si, mas significativamente inferiores aos controles. Enquanto pistas válidas melhoraram o desempenho dos controles e dos pacientes com bvFTD, os pacientes com DA não apresentaram diferença de desempenho entre pistas válidas e inválidas.

**Conclusão:**

Os achados confirmam que os déficits de memória episódica na bvFTD são tão severos quanto na DA e mostram que pacientes com DA, ao contrário dos com bvFTD, não conseguem utilizar efetivamente as informações fornecidas pelas pistas de recuperação, possivelmente em razão de déficits no processamento atencional.

## INTRODUCTION

 The behavioral variant of frontotemporal dementia (bvFTD) features progressive behavioral changes and cognitive deficits, including impaired inhibitory control, attention, and working memory, primarily due to frontal and anterior temporal dysfunction^1-3^. Alzheimer’s disease (AD) is defined by progressive episodic memory impairments from hippocampal atrophy^4^, and episodic memory sparing often differentiates bvFTD from AD. 

 For over a decade, however, it has been shown that bvFTD patients also exhibit episodic memory deficits^5,6^, and in some cases, such deficits can be as severe as those exhibited by AD patients^7,8^. Conversely, for more than two decades, it has been shown that AD may produce significant deficits in executive functions, including deficits in attentional processes^9-11^. Thus, these overlapping impairments raise questions about how executive deficits influence episodic memory in both disorders^6,12-14^. 

 Most bvFTD memory studies used recognition, cued, or free recall tests^7,15-17^ (for a review, see^5^) and a subset of studies used source monitoring tasks^18-20^. Source monitoring tasks involve the identification of contextual details or qualitative aspects of the initial learning phase associated with each test item. For example, individuals may be asked to judge whether words were previously heard in male or female voices or whether pictures of objects were presented on the left or right side of the computer screen^21,22^. Specifically, Söderlund et al.^19^ showed that bvFTD patients exhibited poorer performance than controls in a visual versus aural discrimination source monitoring task, whereas Simons et al.^18^ and Irish et al.^20^ showed that bvFTD were impaired in the source monitoring of spatial and temporal information. Relatedly, several studies found source monitoring deficits in AD, such as deficits in discriminating whether actions were watched or imagined^23^ or discriminating whether words were self-generated or produced by the experimenter. Because activity in prefrontal regions is assumed to be critical for performance in source memory tasks^24^, source monitoring impairments shown by bvFTD and AD patients are thought to be partially caused by prefrontal cortex dysfunctions^18,25^. 

 Source memory tasks, however, are highly heterogeneous regarding the cognitive processes engaged, as well as how heavily they involve executive functions (for a review, see^26^). Thus, here we assessed episodic memory of bvFTD and AD patients through a source monitoring task design to manipulate cognitive control during the spatial retrieval of studied objects^27^. Objects were shown on the left or right side of the screen for study. At test, the same objects were shown on the center of the screen, and participants were asked to report the side where each object was seen before, a task that is largely dependent on medial temporal regions^28-30^. 

 Importantly, however, a portion of the test items were preceded by cues (i.e., hints in the form of arrows pointing to the left or right side of the screen) indicating where on the computer screen each item was previously studied. While most cues were valid (i.e., indicated the correct location) and could be used as supportive evidence for memory decisions, cues that were invalid (i.e., indicated the incorrect location) produced evidence that conflicted with the encoded location, for example, an arrow pointing to the left when the object was previously seen on the right. To produce a correct response in these cases, the participant must respond in opposition to the location hinted by the cue. In the example above, participants must respond "right" to make a correct memory judgment, even though the arrow is pointing to the left. 

 As shown in fMRI studies using an analogous paradigm for recognition memory^31-34^, when participants respond correctly but in opposition to the information conveyed by the cues (i.e., invalid condition), cognitive control brain regions such as the dorsolateral prefrontal cortex and the angular gyrus are engaged^31,32,34^, presumably to resolve the conflict between the incongruent evidence given by the cue and the internal mnemonic evidence produced by the probe. Such cognitive control processes may include inhibitory control to prevent responding according to the information conveyed by the cues^35^, metamemory to assess internal mnemonic evidence before accepting or rejecting cueing evidence^36^, as well as attention and working memory to strategically weigh the strength of internal (memory) versus external (cue) evidence in a manner that can benefit memory performance^34^. 

 In sum, based on prior work examining source monitoring in bvFTD and AD^18-21^ and examining the brain regions engaged during retrieval under cueing^34^, we expected that healthy aging individuals would exhibit greater memory performance than bvFTD and AD in the current spatial memory test. Also, we expected that healthy participants would show the typical cueing effect, with valid cueing increasing memory performance relative to invalid cueing^27^. Such effect, however, should be diminished for AD and bvFTD patients, since they might face difficulties in incorporating the information conveyed by the cues into their memory judgments due to their executive dysfunctions. 

## METHODS

### Participants

 A total of 58 volunteers participated in the study. They were recruited at the university health services of Universidade Federal de Minas Gerais and *Pontifícia Universidade Católica do Rio Grande do Sul*, both located in Brazil. Twenty met consensus criteria for probable behavioral-variant frontotemporal dementia (bvFTD^2^; mean age=67.1, standard deviation [SD]=8.7; 10 females), 18 were diagnosed with probable AD according to Jack et al.^37^ criteria (mean age=74.6, SD=10.2; 9 females), and 20 were healthy controls (HCs) recruited from the community (mean age=63.1, SD=8.0; 12 females). Controls scored within the normal range on the Mini-Mental State Examination (MMSE) using Brazilian norms^38^, and in the clinical interview reported no history of neurological or major psychiatric disorders, denied memory complaints, and were independent in activities of daily living. The current sample sizes were based on a prior study showing strong effects of cueing in parietal and frontal lesion patients^39^. 

 Diagnoses for bvFTD and AD were made by a board-certified neurologist. AD and bvFTD participants underwent brain MRI; individuals with focal lesions or severe vascular disease (Fazekas score ≥2) were excluded. A subset of patients underwent lumbar puncture for biomarker analysis (5/20 bvFTD; 6/18 AD). When available, AD patients showed a biological AD signature defined by the Innotest Amyloid-Tau Index (IATI=Aβ42/(240+1.18 × Tau)) <1; bvFTD patients had IATI >1. For diagnostic accuracy, all patients were followed clinically for at least 24 months, and follow-up diagnoses agreed with baseline classifications for all cases. 

 The study was approved by the Ethics Committee of the Federal University of Minas Gerais (CAAE 17850513.2.0000.5149) and conducted according to the Declaration of Helsinki. All participants provided written informed consent. 

### Materials

 All participants completed the MMSE^38^ and the Frontal Assessment Battery (FAB^40^). Visual episodic memory was assessed with the Figure Memory Test (FMT) from the Brief Cognitive Screening Battery^41^, but because the availability of participants for testing was time-constrained, the FMT was administered only in a subset of the sample (bvFTD n=11; AD n=10; HC n=6). For the source-memory task, 56 object figures were randomly drawn per participant from a 300-item pool and presented on a black screen. Stimuli and responses were controlled with Psychophysics Toolbox for MATLAB (R2007B, v7.5). 

### Procedures

**Figure 1:**
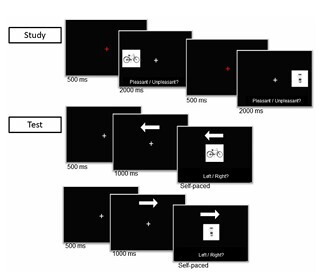
Basic design of the spatial memory task.

 All tests were administered individually. The source-memory task comprised a study phase immediately followed by a test phase. At study, 56 objects were presented in random order, half on the left and half on the right side of the screen. As can be seen in Figure 1, each trial began with a 500 ms fixation cross, then an object appeared for 2 s; participants judged each object as pleasant or unpleasant and gave responses verbally to the experimenter. 

 In the test phase, which was conducted immediately after the study phase, the same 56 objects were shown centrally in random order; participants verbally reported the side where each object had appeared at study ("left" or "right"), and the experimenter recorded responses via keyboard. Seventy-five percent of test trials (42 trials) were cued: an arrow ("←" or "→") appeared at the top of the screen and probabilistically indicated the studied side. Of cued trials, 66.7% were valid (28 trials) and 33.3% invalid (14 trials); the remaining 14 trials were uncued and showed question marks in the cue location. Left/right study locations were balanced across valid, invalid, and uncued trials. Each cue was displayed for 1 s before the object; the cue and object remained on screen until a response. Before the beginning of the experiment, all participants received instructions about how to perform the task and were informed about the frequencies of the valid and invalid cues. In addition, they were informed that they were free to use the cues as hints to improve performance in the memory task. 

### Data analysis

 Because variables were often non-normal, we used nonparametric tests throughout. Group comparisons of age, education, MMSE, and FAB used Mann-Whitney U tests. Baseline (uncued) source memory across groups was assessed with Kruskal–Wallis, followed by Mann–Whitney post-hoc tests. Cueing effects (valid vs. invalid) within each group were tested with Wilcoxon signed-rank tests. Associations between age, education, and cueing effects were tested with Spearman correlations. All tests were two-tailed with α=0.05. Individual data are available at https://osf.io/ca5z7/?view_only=745d6e0cc55b498ea6086c02ca40ed83. 

## RESULTS

### Demographics

 The mean ages and mean years of education of the HC, bvFTD, and AD participants are shown in Table 1. As can be seen in the table, although bvFTDs and HCs had indistinguishable ages, U=146.0, p=0.147, patients with AD were significantly older than both patients with bvFTDs, U=101.0, p=0.022, and HCs, U=69, p=0.001. Also, the HC group had more years of schooling than the bvFTD, U=123, p=0.038, and AD group, U=113, p=0.05, although bvFTD and AD patients had comparable years of schooling, U=176, p=0.920. Patients from the bvFTD and AD groups did not differ in terms of disease duration. 

**Table 1:** Mean scores and p-values for demographic, clinical, and neuropsychological data. Standard deviations are in parentheses.

		HC	bvFTD	AD	HC vs. bvFTD	HC vs. AD	bvFTD vs. AD
Age		63.1 (8.0)	67.1 (8.7)	74.5 (10.2)	p=0.147	p<0.001	p=0.022
Education (years)		14.8 (3.3)	11.8 (4.0)	12.3 (4.9)	p=0.038	p=0.05	p=0.920
Disease duration (years)		—	3.6 (1.7)	3.2 (1.6)	—	—	p=0.496
MMSE		28.6 (1.3)	24.5 (4.0)	24.5 (2.5)	p<0.001	p<0.001	p=0.615
FAB scores							
	Total score	15.7 (1.5)	12.0 (4.3)	12.4 (3.2)	p=0.006	p=0.002	p=1
	Similarities	2.3 (0.7)	1.7 (0.8)	2.0 (0.9)	p=0.048	p=0.418	p=0.327
	Lexical fluency	2.8 (0.4)	1.8 (1.1)	2.2 (0.8)	p=0.003	p=0.015	p=0.285
	Motor series	2.4 (0.9)	2.1 (1.2)	2.1 (1.1)	p=0.645	p=0.429	p=0.818
	Conflicting instructions	3.0 (0.0)	2.2 (1.1)	1.8 (1.3)	p=0.064	p=0.010	p=0.342
	Go/No Go	2.1 (0.8)	1.7 (1.2)	1.7 (1.0)	p=0.280	p=0.219	p=0.920
	Prehension behavior	2.9 (0.2)	2.4 (1.0)	2.8 (0.6)	p=0.267	p=0.749	p=0.748
FMT (5 min)		8.0 (1.3)	6.4 (3.0)	3.7 (1.7)	p=0.246	p=0.001	p=0.014

Abbreviations: HC, healthy controls; bvFTD, behavioral variant of Frontotemporal dementia; AD, Alzheimer’s disease; MMSE, Mini-Mental State Examination; FAB, Frontal Assessment Battery; FMT, Figure Memory Test.

Notes: The FMT was administered in a subsample (bvFTD n=11; AD n=10; HC n=6), while the FAB was administered in all participants, except for 1 HC individual.

### Neuropsychological assessment

 As shown in Table 1, bvFTDs and ADs exhibited indistinguishable scores in the MMSE, bvFTDs versus ADs, U=162.5, p=0.615, although both showed poorer MMSE scores in comparison to HCs [bvFTD x HC, U=51.5, p<0.001; AD x HC, U=21.5, p<0.001]. Similar results were found for the FAB, wherein bvFTDs and ADs showed indistinguishable scores, U=169.5, p=0.771, but both showed inferior scores than HCs [bvFTD x HC, U=93.5, p=0.004; AD x HC, U=71.5, p=0.002]. Because the Figure Memory Test (FMT) was administered to a limited number of participants, we focused only on comparing the two patient groups, a comparison that showed a memory advantage for bFTD relative to AD patients, U=19.5, p=0.014. Thus, as expected, HC showed greater performance than both bvFTDs and ADs in the neuropsychological tests, while the patient groups showed equivalent performances on the MMSE and on the FAB. Interestingly, bvFTD patients showed greater performance on a visual memory test compared to AD patients. 

### Spatial retrieval performance

#### Baseline performance

**Figure 2:**
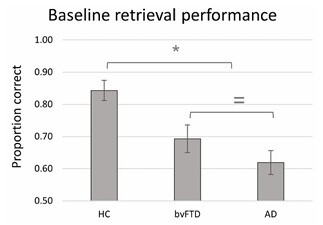
Baseline retrieval performance for each group.

 As can be seen in Figure 2, the baseline source memory performances shown by HC, bvFTD, and AD participants were apparently distinct, a pattern that was confirmed by the statistical analysis, H (2)=14.79, p<0.001. Follow-up pairwise comparisons showed that HC individuals showed a significant advantage in comparison to patients with bvFTD, U=295, p=0.010, r=0.47, and AD, U=304, p<0.001, r=0.69. The apparent advantage shown by bvFTD relative to AD patients, however, was not significant, U=133, p=0.175, r=0.26. 

 Thus, as in prior reports, individuals with bvFTD and AD exhibited impaired episodic memory relative to healthy controls. When directly compared, however, individuals with bvFTD and AD showed statistically indistinguishable source memory performances. The same effects were found (omnibus and posthoc) when these data were analyzed with an ANCOVA including age as a covariate, but for consistency, we report only the non-parametric results. 

#### Retrieval under cueing

**Figure 3:**
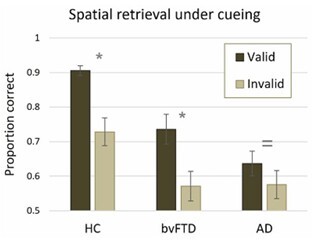
Retrieval of spatial information under cueing.

 To investigate whether source memory for spatial location was affected by cueing, we analyzed the memory performances of each group of participants for objects preceded by valid versus invalid cueing (Figure 3). Both HC and individuals with bvFTD showed significant cueing effects, with valid cues producing significantly greater performances than invalid cues (HC, W=178, p<0.001, r=0.874; bvFTD, W=119, p=0.047, r=0.556). Individuals with AD, however, showed no reliable effect of cueing, W=95, p=0.162, r=0.404, exhibiting indistinguishable performances after valid versus invalid cues. 

 There was no significant correlation between the age of the AD patients and their validity effects (valid minus invalid performance, r=-0.15, p=0.563). Thus, although the AD patients were on average older than participants from the other groups, age does not seem to underlie the current absence of cueing effects. Correlations between age and validity were also non-significant for bvFTD, r=0.37, p=0.112, and healthy controls, r=-0.17, p=0.482. Finally, no significant correlation between cue validity and education was found (AD, r=0.42, p=0.080; bvFTD, r=-0.04, p=0.868; HD, r=-0.36, p=0.120). 

## DISCUSSION

 BvFTD and AD patients showed impaired baseline performances compared to HCs in the spatial source memory task. Direct comparison between bvFTD and AD revealed no between-group differences, consistent with prior studies showing indistinguishable memory performances between these groups^6^ accompanied by strong deficits relative to healthy individuals^18-24^. Unexpectedly, while HC and bvFTD responses were similarly affected by cueing, AD responses were not—cues exerted no effects on AD performance. 

 Considering baseline performance on the spatial task, the marked impairments in bvFTD and AD relative to healthy controls, and the lack of difference between the patient groups, support the view that episodic memory is an unreliable discriminator between these dementias, extending this conclusion to spatial-memory tests^6^. This aligns with evidence that bvFTD can produce episodic deficits as severe as AD^5,7,8^. Given the spatial demands, shared deficits may reflect common neural substrates (e.g., medial-temporal dysfunction^28-30^), warranting neuroimaging investigation. BvFTD patients showed better FMT than source task performance, although this is tentative given limited sample sizes (bvFTD n=11; AD n=10). 

 The absence of cueing effects for AD suggests these patients, unlike HC and bvFTD, could not properly attend to cue information while performing memory judgments. This might result from attentional deficits^10,42,43^, with consequences for spatial retrieval and orientation in AD^44^. AD produces attentional process deficits even in early stages^45,46^, including inhibitory control and spatial attention deficits^47^. AD also produces spatial orientation deficits^48^, found in early stages^49^ and considered a potential early detection index^44^. 

 Since the task involves attending to cues orienting participants toward previous item locations, it presumably relies on both attention and spatial orientation mechanisms. These findings suggest AD-related spatial orientation deficits^48^ might partially result from attentional mechanisms that preclude attending to environmental orienting cues. For example, impaired ability to attend to salient cues (e.g., white coats in a doctor’s office) may cause difficulties judging location when asked "where are you now?" Though hypothetical, this encourages investigating attentional deficits’ role beyond memory deficits in AD spatial orientation impairments. 

 BvFTD patients showing cueing effects analogous to healthy individuals was unexpected, considering that cue information incorporation engages cognitive control regions^31,32,34^. The cognitive control deficits often exhibited by bvFTD are apparently not detected by current cueing procedures, though baseline performance showed severe spatial retrieval deficits. The task confirms memory impairment in bvFTD but leaves unanswered whether executive dysfunctions contribute to such deficits. 

 Taken together, the current findings add support for the notion that individuals with both bvFTD and AD have significant episodic memory deficits. Furthermore, it suggests that AD patients have difficulties in attending to environmental cues while performing tasks involving spatial retrieval. Further studies should investigate the role of attention in the deficits shown by AD in spatial orientation tasks, a research avenue that can lead to the development of screening batteries with greater sensitivity to detect early-stage AD. 

### Limitations

 Limitations include a small sample size that precludes parametric analyses and limits statistical power. The lack of biological investigation with cerebrospinal fluid biomarkers of all patients from bvFTD and AD groups hampers the accuracy of the clinical diagnosis. We did not use clinically validated scales for disease staging of bvFTD and AD, which would allow a more valid comparison of disease stage and progression between clinical groups. The restriction of the FMT to a subsample precluded analysis focusing on bvFTD patients with different cognitive phenotypes. Finally, although invalid cueing during retrieval consistently engages cognitive control regions^31,32,34^, future functional magnetic resonance imaging studies should verify whether bvFTD and AD patients show similar activations. Such work could clarify executive control’s role in memory retrieval. Despite these limitations, our study advances understanding of spatial memory retrieval under orienting cues in bvFTD and AD and may guide development of cognitive tools to distinguish these disorders in clinical practice. 

## Data Availability

The datasets generated and/or analyzed during the current study are publicly available at Open Science Framework (https://osf.io/ca5z7/?view_only=745d6e0cc55b498ea6086c02ca40ed83).
